# Adapting Psychiatric Approaches to the Needs of Vulnerable Populations: A Qualitative Analysis

**DOI:** 10.3390/ejihpe15030030

**Published:** 2025-02-28

**Authors:** Pascale Besson, Lison Gagné, Bastian Bertulies-Esposito, Alexandre Hudon

**Affiliations:** 1 Department of Psychiatry and Addictology, Faculty of Medicine, Université de Montréal, Montreal, QC H3T 1J4, Canada; pascale.besson@umontreal.ca (P.B.); lison.gagne.ccsmtl@ssss.gouv.qc.ca (L.G.); bastian.bertulies-esposito@umontreal.ca (B.B.-E.); 2 Centre de Recherche de l‘Institut Universitaire en Santé Mentale de Montréal, Montreal, QC H1N 3V2, Canada

**Keywords:** psychiatry, mental health, clinical competence, vulnerable population, social justice, Housing First, intersectoral collaboration, needs assessment

## Abstract

Marginalized populations face significant barriers to mental health care, such as stigma, poverty, and limited access to adapted services, with conventional psychiatric approaches often falling short. This study aimed to explore how psychiatric care can be adapted to better meet the needs of vulnerable populations. Data were collected from psychiatry residents, psychiatrists, and community organization staff during a course on vulnerable populations, using semi-structured discussions and analyzed through grounded theory with iterative coding. Seven main themes emerged: (1) barriers and needs of vulnerable populations, highlighting challenges like homelessness and stigma; (2) psychiatric interventions and flexible approaches, emphasizing tailored care; (3) collaboration with community organizations, focusing on partnerships to improve care access; (4) ethical approach and respect for rights, ensuring dignity in treatment; (5) specific populations and associated challenges, addressing the needs of groups like LGBTQ+ youth and migrants; (6) intervention and support models, such as proximity-based care and post-hospitalization follow-up; (7) innovation and evolution of practices, focusing on research and institutional adaptations. This study emphasizes the need for personalized, intersectoral care, recommending improved collaboration, flexible models, and greater clinical exposure, with future research exploring how psychiatric education can better prepare clinicians to work with marginalized groups.

## 1. Introduction

Marginalized populations, such as those experiencing homelessness, migrant populations, ethnic minorities, LGBTQ+ individuals, and people living with severe mental health disorders, have specific needs that are often insufficiently considered. These groups encounter socio-economic barriers like stigma, poverty, and limited access to healthcare, worsening their mental health (Galea et al., 2011; Magwood et al., 2020). However, conventional approaches are often not adapted to the specific needs of marginalized populations, who required adjustments in the therapeutic approach, care delivery settings (Farkas et al., 2016), and psychological support. Healthcare systems often lack the specialized training and resources to meet these needs effectively (Baxter et al., 2019; Farkas et al., 2016). For instance, individuals experiencing homelessness, with complex psychiatric issues, struggle to access integrated care, which exacerbates their vulnerability (Hwang et al., 2011). They are at high risk of re-hospitalization due to the complexity of their needs and unstable living situations (Russolillo et al., 2023).

Adapting mental health interventions to the life contexts and cultural particularities of these populations is essential to improving clinical outcomes and quality of life (Kirmayer & Pedersen, 2014). Community-based initiatives that strengthen social networks and create support networks are particularly effective in reducing inequalities and promoting better mental health (Castillo et al., 2019). By collaborating with local organizations and involving users directly, solutions can be tailored to meet the specific needs of communities, creating more inclusive and accessible environments (Stergiopoulos et al., 2014). 

To address these specific needs, innovative models like integrated services combining mental health and housing have proven effective. Barker et al. (2022) and Wang et al. (2019) highlight the importance of collaborative approaches, particularly for homeless youth. These initiatives, which include interventions such as cognitive-behavioral therapy and the “Housing First” model, simplify access to care, enhance residential stability, and improve mental health and substance use outcomes. By focusing on patients’ living conditions, these models encourage engagement in care and reduce socio-economic barriers. 

Cultural differences influence perceptions of mental illness and treatment, which can affect the therapeutic relationship and patient adherence (Bhui & Morgan, 2007). Adapting interventions to cultural realities enhances their effectiveness and patient engagement, especially among refugees and other marginalized groups (Fennig, 2021). Psychiatric interventions must also be sensitive to patients’ socio-economic contexts and designed to overcome organizational barriers that limit their impact (Daniels et al., 2022). Effectiveness relies on a flexible, integrated approach tailored to the specific needs of populations while utilizing available resources. To reduce disparities, inclusive solutions must be implemented that improve accessibility and develop structural competence in clinicians, enabling them to respond to systemic inequalities and adapt their practices to institutional and social dynamics (Hatzenbuehler et al., 2018). Structural competence refers to healthcare professionals’ ability to recognize, analyze, and address social, economic, and institutional structures that influence health and inequalities (Metzl & Hansen, 2014).

Despite these particular needs and barriers to care experienced by vulnerable populations, psychiatric training for medical residents seems insufficient in preparing them to work with these groups (Cooke et al., 2013), notably due to a lack of clinical exposure (Olfson et al., 2021). Therefore, it is recommended to integrate modules focused on social determinants and to increase rotations opportunities with disadvantaged populations (Williams et al., 2020). Sudak et al. (2020) focus on the importance of raising residents’ awareness of implicit biases and inequalities, incorporating simulations and diverse case studies. The COVID-19 pandemic has amplified these challenges for clinicians from different fields, with resource shortages and time constraints making vulnerable populations even more invisible, highlighting the need for cultural sensitivity and a better understanding of social determinants of health (Marsh et al., 2020).

Corrigan and Al-Khouja (2018) show that programs integrating community center internships and training on stigma improve residents’ attitudes and enhance their clinical skills. These educational experiences provide a comprehensive, inclusive understanding of mental health, helping reduce inequalities in access to psychiatric care. To move forward, it is crucial to consider the perspectives of psychiatric residents and community workers, who play a key role in improving care for these populations. 

This study seeks to answer the following question: *How should psychiatric care be adapted to better address the needs of vulnerable and marginalized populations?* We sought to identify components of interventions that lead to enhanced care for vulnerable populations. To meet this goal, the following specific objectives were defined: to identify the main challenges faced by clinicians in adapting psychiatric care for these groups, to analyze the benefits and obstacles perceived by psychiatry residents and community agency workers regarding adjustments in the psychiatric approach, and lastly, to provide recommendations to improve the accessibility and quality of psychiatric care for these specific populations.

Based on the available data in the current literature, several hypotheses can be formulated: clinicians and residents perceive systemic inequalities and lack of resources as significant obstacles to adapting psychiatric care for vulnerable populations. Additionally, many clinicians and residents lack training in structural competence, which limits their ability to address the unique needs of these groups effectively. Specific training programs and collaboration with community organizations are often viewed as beneficial in overcoming these challenges. However, organizational constraints, such as heavy workloads and restrictive institutional policies, are identified as major barriers to integrating new psychiatric practices. Finally, promoting interdisciplinary programs that support collaboration between mental health professionals and social workers is seen as a promising approach to address the needs of vulnerable populations.

## 2. Materials and Methods

### 2.1. Participants

The participants included PGY2 psychiatry resident physicians (trainees who have completed doctoral studies in medicine) at the Université de Montréal and psychiatrists affiliated with the Université de Montréal, as well as mental health professionals from the Old Brewery Mission, a community organization, the Intensive Support for Homelessness Program (SII), and the Project for Reaffiliation in Homelessness and Mental Health (PRISM). The mental health professionals were social workers, nurses, specialized educators, and psychosocial support workers. All participants are engaged in providing care and support to vulnerable and marginalized populations in their respective workplaces. It should be noted that they all work in an urban setting. The resident physicians were recruited during their course on vulnerable populations and clienteles, as part of their second year of residency, while the mental health professionals and psychiatrists were recruited through their involvement in this course. The recruitment followed a convenience sampling approach, with participant selection carried out by LG and PB. No invited participants declined to take part in the project. This course took place in August 2024 and lasted a day. This project was approved by the Université de Montréal Ethics Committee (approbation number 2024-6385). 

### 2.2. Data Collection

Data were collected through routine recordings made during the course on vulnerable populations (these recordings are typically available on a website available to psychiatry residents) and are uploaded for the benefit of the resident physicians. Data were gathered from lectures and free-flowing, semi-structured discussions conducted within the course, involving the expertise of multiple mental health professionals from different fields. The course focused on the specific needs of vulnerable populations, the challenges encountered in adapting psychiatric approaches, and participants’ perceptions of the benefits and limitations of the adjustments applied during the course. The course was conducted over the span of a full day (Table 1). The triangulation of data was carried out by using different sources to explore the same phenomenon. No participant corrected or clarified any aspect of the interview. The COREQ report was followed. The criteria of credibility, transferability, dependability, and confirmability were applied in this study to ensure its rigor as per Lincoln and Guba (1985).

### 2.3. Data Analysis

After transcription of the recordings, themes were identified from the transcript using the grounded theory framework (Chun Tie et al., 2019). A preliminary coding scheme based on these themes was developed by PB and AH and applied to the transcript using Qualitative Data Analysis Miner software (QDAMiner 6) (Chomczynski, 2008). The annotations made by PB and AH were then compared, and interrater agreement was assessed using Scott’s Pi, a measure that quantifies the consistency among raters (indicating the level of consensus or homogeneity in their evaluations). 

Differences between the coders were used to refine the list and definitions of themes. This involved splitting existing themes, consolidating codes under broader themes, adding new themes, or removing redundant ones. The updated theme list was then re-applied to the transcript using QDA Miner. This iterative process continued until the Scott’s Pi reached an acceptable level and data saturation was achieved. Acceptable agreement levels were defined based on the SAGE Research Methods benchmark, where a Scott’s Pi of 0.81–1.00 indicates almost perfect agreement, 0.61–0.80 substantial agreement, 0.41–0.60 moderate agreement, 0.21–0.40 fair agreement, 0.0–0.20 slight agreement, and less than 0 poor agreement (Scott, 2017).

During the first iteration, with 9 themes and 39 sub-themes applied to the transcript, a Scott’s Pi of 0.71 was achieved. After refining the themes and sub-themes, reducing the number to 7 and 31, respectively, the Pi score improved to 0.82. In the final iteration, after further adjustments to theme definitions, PB and AH achieved a Scott’s Pi reflecting an almost perfect agreement. 

## 3. Results

The data collected, which led to the following results, come from open, semi-structured discussions among 12 s year psychiatry residents from the Université de Montréal, 3 expert psychiatrists teaching at the Université de Montréal, and 11 mental health professionals working in the community, as part of a course offered to residents. The total recording time for the courses is 6 h, 25 min, and 54 s. A total of 4582 sentences constitutes the full verbatim transcript of the courses.

**Figure 1:**
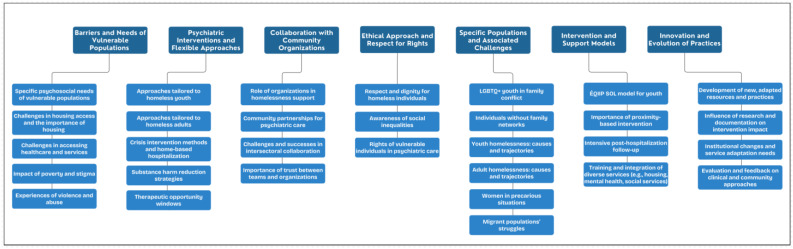
Thematic codification of adaptations in psychiatric approaches to address the needs of vulnerable populations and specific clientele. The main themes are highlighted in dark blue, while the sub-themes are presented in light blue.

Seven themes emerged from data analysis, composed of 31 sub-themes, as shown in (Figure 1). Table 2 provides further information on sub-themes, and examples are provided for each of these.

### 3.1. Barriers and Needs of Vulnerable Populations

It refers to the challenges such as homelessness, poverty, or stigma faced by individuals in vulnerable groups. Here are different sub-themes that emerged from this theme.

#### 3.1.1. Specific Psychosocial Needs of Vulnerable Populations

This sub-theme refers to the mental, emotional, and social needs that individuals from marginalized groups face. Examples from the courses:

*Mental health professional A:* “You need to have filed your taxes and have ID (to access Subsidized Housing), but when you’re homeless, you have none of that”.

*Psychiatrist A:* “You might spend a few days in detention at Bordeaux or Leclair (detention centers) and have to complete all your applications for social assistance, which is cut off from the start (of detention)”.

#### 3.1.2. Challenges in Housing Access and the Importance of Housing

Housing access challenges highlight the various barriers preventing individuals from securing stable and affordable housing, including financial constraints, discrimination, and limited availability of suitable housing options. The importance of housing underscores how stable housing is essential for overall well-being, providing a foundation for individuals to rebuild their lives and access necessary resources. Examples:

*Psychiatrist B:* “When we talked about developing two new (Project for Reaffiliation in Homelessness and Mental Health) PRISM, I thought, there’s no point in developing new PRISM if you don’t have housing options to back them up”.

*Mental health professional B:* “For regular public housing, it’s an 8- to 10-year wait (for access)”.

#### 3.1.3. Challenges in Accessing Healthcare and Services

Various challenges in accessing healthcare and services refer to obstacles that can prevent individuals from receiving care that meets their specific needs. Examples:

*Resident A:* “What is also interesting is that these services work as long as we have a strong social safety net, because if the person is not ready to access your services because they don’t want to, they are not willing at that moment, we still need to have some assurance that between that moment and the moment they are ready, they might have access to income, to social assistance, that they won’t just completely fall into nothingness”.

*Psychiatrist B:* “That’s why I try to say that the waiting list is like the worst thing, because we will lose this ability to be a kind of community presence that can act at the right moment”.

#### 3.1.4. Impact of Poverty and Stigma

The impact of poverty and stigma refers to how socioeconomic disadvantage and societal discrimination can negatively affect mental health and access to resources. Examples:

*Psychiatrist A:* “And sometimes, we’re told… there’s a lot of resistance to accepting our patients (patients with mental health conditions) into services, even outpatient ones, when we try to pass the baton”.

*Psychiatrist B:* “Because if, in society, we have all these cognitive biases against homeless people, there is no pressure on the system to organize sensible services—no pressure at all”.

#### 3.1.5. Experiences of Violence and Abuse

This sub-theme emphasizes how violence and abuse can deeply affect vulnerable individuals, often intensifying mental health problems and complicating their ability to seek help or stability. Examples:

*Psychiatrist B:* “More often they’re victims, though some commit crimes too, but more often they’re victims *(homeless individuals)*”.

*Mental health professional D:* “I had a patient who was assaulted by security officers, on the ground, with really serious injuries. It was a whole ordeal, all because she responded, gave some attitude, and kind of insulted the person”.

### 3.2. Psychiatric Interventions and Flexible Approaches

This theme focuses on providing comprehensive, patient-centered care that aligns with diverse needs and changing contexts. Five sub-themes came out of it.

#### 3.2.1. Approaches Tailored to HOMELESS Youth

Those approaches focus on addressing the needs of young people experiencing homelessness. Examples:

*Psychiatrist C:* “We really try to ensure that there are no wrong entry points: the young person chooses the organization that suits them best”.

*Psychiatrist C:* “For youth, this (a group home) is useful because there are many, as I’m telling you, especially those coming out of youth protection services—often, they don’t know how to cook or do other basic tasks, and group homes help them acquire these skills”.

#### 3.2.2. Approaches Tailored to Homeless Adults

Similarly, those approaches aim to tackle challenges like mental health disorders, substance use, and housing instability, with strategies designed specifically for adults. Examples:

*Psychiatrist B:* “We want certain things; they want certain things, and sometimes we have to accept that we don’t necessarily want the same things”.

*Psychiatrist B:* “For our patients who lose housing, we bring them to the social worker, work on housing options, guide them, and maintain the connection”.

#### 3.2.3. Crisis Intervention Methods and Home-Based Hospitalization

This sub-theme emphasizes immediate mental health support, offering care in less restrictive environments such as the individual’s home to promote comfort and accessibility. Examples:

*Psychiatrist B:* “Versus having options where, as soon as a problem is identified, a team can come to the home, providing support to preserve what’s left—keeping the job, making sure all financial support is available to help through this period without losing too much”.

*Psychiatrist B:* “The more flexibility we have, the better we can adapt to the person’s needs, and the efficiency of our interventions increases as well”.

#### 3.2.4. Substance Harm Reduction Strategies

Substance harm reduction strategies focus on reducing the negative effects of substance use through safer practices, access to clean supplies, and supportive services, rather than solely aiming for abstinence. Examples:

*Mental health professional E:* “Despite this, we emphasize tolerance and harm reduction, so OBM-Pavillon Webster, for example, distributes syringes, injection liquids, crack pipes, meth pipes, and so on”.

*Psychiatrist B:* “But I tell myself that I will work on a variety of non-specific factors related to consumption, knowing that these improvements in the conditions in which the brain functions will likely lead to a cascade of positive effects, including reduced substance use for various reasons”.

#### 3.2.5. Therapeutic Opportunity Windows

This sub-theme refers to critical moments in an individual’s life when they are more receptive to treatment or behavioral changes. Examples:

*Mental health professional F:* “When they (the patients) knock on the door (of PRISM-Webster) for help, we (the PRISM team) don’t put it off until next week; it’s now”.

*Psychiatrist B:* “But we try to provide the best possible chances with the opportunities we have”.

### 3.3. Collaboration with Community Organizations

It was made clear during the conversations between professionals that this partnership between healthcare providers and local groups improves access and addresses social needs. Here are the four sub-themes associated.

#### 3.3.1. Role of Organizations in Homelessness Support

The role of organizations in homelessness support highlights how non-profits, community groups, and other entities provide critical services such as housing assistance, healthcare, and vocational training to individuals experiencing homelessness. Examples:

*Mental health professional A:* “If Diogène hadn’t been there, we would never have been able to find housing for a woman with her three children”.

*Psychiatrist B:* “OBM (Old Brewery Mission) has really shifted toward the goal of exiting homelessness, whereas before, it was a shelter focused on keeping people alive. But they developed their services, with housing and programming, so if people stay there, it’s because they accept the concept of working toward exiting homelessness”.

#### 3.3.2. Community Partnerships for Psychiatric Care

Those partnerships emphasize collaborative efforts between mental health providers and community organizations to offer integrated, holistic care tailored to the needs of vulnerable populations. Examples:

*Psychiatrist A:* “Sometimes, in emergency situations, we receive patients who have been referred for psychiatric evaluation by community organizations”.

*Psychiatrist B:* “The basis of collaboration is recognizing that we are powerless to handle things alone”.

#### 3.3.3. Challenges and Successes in Intersectoral Collaboration

This sub-theme refers to the obstacles, such as communication gaps and differing priorities, as well as the achievements that arise when sectors like healthcare, housing, and social services work together to address complex issues. Examples:

*Mental health professional C:* “When you’re in a 24-7 environment with 12 people who have observed the same thing, and you send the person for consultation, and they tell you the person is fine, you’re like: I’m not a psychiatrist, but no, they’re not fine, I’m telling you”.

*Psychiatrist A:* “Community organizations report experiences where they send people, say, to the hospital or emergency room with great concern, and they’re sent right back without consultation”.

#### 3.3.4. Importance of Trust Between Teams and Organizations

The importance of trust between teams and organizations underlines how strong relationships and mutual trust among service providers are vital for effective collaboration and consistent support for those in need. Examples:

*Psychiatrist A:* “And then, after all the efforts made by the organization, they see the person reappear without any prior notice from the hospital”.

*Resident C:* “We don’t want to disrespect the people who referred the individual to you, who did incredible work”.

### 3.4. Ethical Approach and Respect for Rights

The theme “Ethical approach and respect for rights” highlights the importance of upholding individuals’ dignity, autonomy, and fundamental rights. Three following sub-themes emerged from this theme: 

#### 3.4.1. Respect and Dignity for Homeless Individuals

This sub-theme emphasizes the importance of treating people experiencing homelessness with honor and compassion, recognizing their humanity and fundamental rights. Examples:

*Psychiatrist B:* “If you simply dismantle the camps, remove their tents, throw everything away, and disperse them, you’re acting against their rights”.

*Psychiatrist B:* “If you can provide them with a clean, fresh space, bread, and minimal amenities, you’re operating on a whole different level when it comes to human dignity”.

#### 3.4.2. Awareness of Social Inequalities

Awareness of social inequalities is a sub-theme that focuses on understanding and addressing the systemic disparities that disproportionately impact vulnerable groups, including access to resources, healthcare, and opportunities. Examples:

*Psychiatrist A:* “And she *(a patient)* was released from her sentence of over two years in Montreal; she had never even set foot in Montreal before!”

*Psychiatrist A:* “This year, there is a project underway to support internal medicine residents from CHUM at Maison du Père”.

#### 3.4.3. Rights of Vulnerable Individuals in Psychiatric Care

This sub-theme underlines the legal and ethical obligations to ensure fair, equitable, and respectful treatment of marginalized populations within mental health services, safeguarding their autonomy and well-being. Examples:

*Psychiatrist B:* “The entire impact, the burden of illness—if we can ease it by reducing social stigma related to illness, by allowing the person to feel a sense of control over their life, a feeling of capability, because nothing is as disempowering as being on a psychiatric ward, in a hospital gown, without your cell phone, unable to do anything”.

*Mental health professional A:* “So, she’d *(a client)* leave for four weeks, make the rounds, come back, and everyone would say, well, she’s odd, but no one called, no one reached out for help, because, well, “that’s life, that’s how she is, we have to respect that”… but people also have the right to receive care, you know”.

### 3.5. Specific Populations and Associated Challenges

“Specific populations and associated challenges” is a crucial theme that refers to groups of individuals facing distinct difficulties due to factors such as socioeconomic status, mental health, sex, sexual orientation, gender, or homelessness. Seven sub-themes came out of it: 

#### 3.5.1. LGBTQ+ Youth in Family Conflict

This sub-theme explores the unique struggles faced by young people in the LGBTQ+ community who experience discord with their families, which can lead to homelessness, mental health challenges, and a lack of support. Examples:

*Psychiatrist C:* “It’s important to think about this *(discrimination within the family)*, because often we may want them to return to their family”.

*Resident D:* “…the proportion *(of homelessness)* of people from LGBTQ communities was very high, precisely because they unfortunately faced more family conflicts that could lead to being kicked out”.

#### 3.5.2. Individuals Without Family Networks

This sub-theme refers to people who lack supportive familial connections, leaving them more vulnerable to instability and difficulties in accessing resources. Examples:

*Resident D:* “At a symptomatic level, it has to get worse before someone takes you to *services (when the person has no family or social network)*”.

*Resident D:* “Prolonged cases of untreated illness, greater losses, increasing disorganization—therefore, steeper climbs to recovery and potentially violent or dangerous situations for patients and others *(when the person has no family or social network)*”.

#### 3.5.3. Youth Homelessness: Causes and Trajectories

These causes and trajectories explore the factors that contribute to homelessness among young people, as well as the different paths they may take during their experience of homelessness. Examples:

*Psychiatrist C:* “And often, what we see is young people who have come out of child protective services (DPJ), and now they want to reunite with their family, but it’s the same families that were inadequate in the first place”.

*Psychiatrist C:* “So, often, young people who have been expelled may have been kicked out because of behaviors, such as violence or substance use, which can be difficult for the family to handle. But there are also many young people who flee their families because of abusive situations”.

#### 3.5.4. Adult Homelessness: Causes and Trajectories

Similarly, these causes and trajectories focus on the reasons behind homelessness in adults and the varied experiences they may encounter. Examples:

*Psychiatrist B:* “You know, it’s like they come from a caste of misery, but in reality, you find out there were plumbers, property owners, a family doctor, a psychologist, a circus artist, fathers, mothers”.

*Psychiatrist B:* “There are also people from broken families who have been homeless since youth, who encountered drugs so early that they couldn’t go through normal development”.

#### 3.5.5. Women in Precarious Situations

This sub-theme highlights the specific challenges faced by women in unstable living conditions, including issues like domestic violence, homelessness, and economic insecurity. Examples:

*Mental health professional C:* “In fact, for many women, it only takes a situation of financial instability and the weakening of their social network for them to end up here *(at OBM-Women)*”.

*Mental health professional A:* “Mental health in women presents very differently than in men”.

#### 3.5.6. Migrant Populations’ Struggles 

The struggles faced by migrant populations, such as immigration instability, language barriers, and limited access to resources, were brought up during the courses. Examples:

*Resident B:* “This reminds me of people who live with immigration instability, who have no status, who must apply for asylum, who might be here without any legal status—like ghost visitors”.

*Mental health professional C:* “Now, we really see cases of homelessness appearing at age 50, the first experience… women who are unfamiliar with it, who have no history of substance use, who are asylum seekers, who have just arrived in the country, who don’t speak the language”.

### 3.6. Intervention and Support Models

This theme refers to structured approaches and strategies designed to address the needs of individuals facing challenges such as homelessness, mental health issues, or social instability. Here are the four sub-themes related:

#### 3.6.1. Quebec Intensive Psychiatric Intervention Team—Support in Living Environments (ÉQIIP SOL) Model for Youth

The ÉQIIP SOL model for youth is a Montreal-based program providing intensive psychiatric care to youth with severe mental health disorders and social vulnerabilities, such as homelessness. Examples:

*Psychiatrist C:* “These young people have access to all the services at the JAP Clinic *(First psychosis episode clinic)*, but what they get in addition is support from workers who are more available”.

*Psychiatrist C:* “The workers have half the caseloads of others and also routinely spend time in community organizations”.

#### 3.6.2. Importance of Proximity-Based Intervention

This sub-theme underscores the value of delivering care and support close to where people live, making services more accessible and fostering better engagement. Examples:

*Psychiatrist B:* “But we keep the relationship, we stay connected, we continue helping them as they go, until their passing if needed, which is the palliative approach, or maybe there will be a turning point”.

*Psychiatrist A:* “Just being present has radically changed the person’s path and trajectory… it’s amazing how people accept care here *(in the community)* that they’d never accept in other circumstances”.

#### 3.6.3. Intensive Post-Hospitalization Follow-Up

Post-hospitalization follow-up focuses on ensuring continuity of care after individuals leave the hospital, reducing the likelihood of relapse or readmission through ongoing support and monitoring. Examples:

*Psychiatrist C:* “An approach focused on individuals with substance use disorders involves providing early and intensive follow-up, being highly active and proactive in post-hospitalization care and during crises through crisis intervention and incorporating a strong housing support component”.

*Resident E:* “If she *(a patient)* becomes dangerous and you believe hospitalization is necessary, could she return to PRISM here afterward, somewhat like a TIBD model but under PRISM?”

#### 3.6.4. Training and Integration of Diverse Services

Lastly, training and integration of diverse services, such as housing, mental health, and social services, emphasizes the importance of equipping service providers with comprehensive training and coordinating efforts across sectors to address the complex, interconnected challenges faced by vulnerable groups. Examples:

*Psychiatrist B:* “There’s also the concept of convalescence beds, which are collaborations between internal medicine doctors and shelters, where shelters handle, for instance, IV antibiotics, when there’s an IV antibiotics course, or when there’s a PICC line”.

*Psychiatrist B:* “For example, we have agreements with RAMQ so we can issue a RAMQ card ourselves *(at the Intensive Support for Homelessness Program: SII)*”.

### 3.7. Innovation and Evolution of Practices 

“Innovation and evolution of practices” is the final theme that came out of the courses. This theme refers to the ongoing development and refinement of mental health approaches, driven by advancements in research, technology, and understanding of social determinants of health. The four following sub-themes are connected to this theme: 

#### 3.7.1. Development of New, Adapted Resources and Practices

This sub-theme focuses on creating innovative tools and methodologies tailored to the evolving challenges faced by these communities. Examples:

*Psychiatrist A:* “The SII team is large enough to do outreach and move around in the metro stations, in the shelters where they’re staying”.

*Psychiatrist B:* “So, the idea initially was to create a reaffiliation program *(with PRISM and SII)*”.

#### 3.7.2. Influence of Research and Documentation on Intervention Impact

This sub-theme underscores how empirical studies and evidence-based insights shape effective interventions and improve service delivery. Examples:

*Psychiatrist A:* “For information, Médecins du Monde is currently conducting a research project to document mental health practices for homeless populations, which includes cities beyond Montreal, aiming to broaden the scope and share knowledge”.

*Psychiatrist B:* “This also allows us to calculate the number of people transitioning out of homelessness each year”.

#### 3.7.3. Institutional Changes and Service Adaptation Needs

Institutional changes and service adaptation needs highlight the necessity for organizations to adjust and innovate their services in response to shifting societal demands and specific vulnerabilities. Examples:

*Psychiatrist B:* “There are public health aspects, there are entrepreneurial aspects, but in fact, we’re not here just to provide medical services; we’re here to be part of a project to solve a social problem”.

*Psychiatrist B:* “The government included PRISM in its action plan for reducing homelessness”.

#### 3.7.4. Evaluation and Feedback on Clinical and Community Approaches

Lastly, evaluation and feedback on clinical and community approaches is a sub-theme that stresses the role of ongoing assessment and stakeholder input in refining practices to ensure their relevance and effectiveness. Examples:

*Resident F:* “I think the disorganized aspect allows for flexibility and adapts to needs”.

*Psychiatrist A:* “It gives validity to the evaluation, it improves the validity of your evaluation when you’re in the person’s environment, and it allows you to better tailor your intervention to genuinely meet their needs”.

## 4. Discussion

This study’s findings revealed several important perceptions from residents, psychiatrists, and other mental health professionals regarding the adaptation of mental health care for vulnerable populations. Participants emphasized the importance of housing stability and socio-economic factors. They highlighted the barriers these populations face to accessing appropriate care. They also noted the need for a flexible and intersectoral approach to address the complex needs of people experiencing homelessness and other marginalized individuals. These findings align with elements already present in the literature, particularly on issues related to access to care and tailored approaches for each situation (Barker et al., 2022; Wang et al., 2019).

When discussing barriers and needs of vulnerable populations, residents, psychiatrists, and community mental health workers highlighted that the psychosocial needs of those populations are multifaceted and complex, particularly due to structural barriers like access to housing and care. The literature also indicates the importance of these factors for the mental health of individuals, and the connection between housing and mental well-being has been widely explored. For instance, Padgett (2020) and Mejia-Lancheros et al. (2020) demonstrated that housing instability and social stigma are significant factors that hinder access to mental health care. Socio-economic factors such as poverty and stigma were identified as major barriers to care by participants. This observation aligns with several studies that show the critical role housing plays in emotional and mental stability (Baxter et al., 2019).

Regarding psychiatric interventions and flexibility of approaches, the results indicate that mental health workers recognize the need to adapt psychiatric approaches for different populations, including youth and adults experiencing homelessness. Crisis intervention methods, such as home hospitalization, are seen as more humane and appropriate, especially in vulnerable contexts. Experts also emphasized the importance of taking advantage of therapeutic opportunity windows and harm reduction strategies. The literature also recommends flexible approaches to crisis intervention, such as mobile psychiatric services and harm reduction for substance users (Pinkhover et al., 2024). The emphasis on flexibility and early intervention in the study supports trends observed in research on the importance of rapid access to care and the growing body of evidence advocating/demonstrating the need for early intervention in mental health (Castle et al., 2018; McGorry et al., 2024; Uhlhaas et al., 2023).

When addressing the collaboration with community organizations, participants emphasized the importance of those partnerships to support vulnerable populations. Trust and communication between teams and organizations are essential for effective care. The literature on intersectoral intervention and collaboration also highlights the impact of these dynamics in improving access to health services and other essential resources, particularly for marginalized populations (Kloos et al., 2021). These partnerships can reduce administrative and social barriers, as shown in Tsemberis’s (2010) work on the Housing First model, which demonstrates the positive impact of collaboration between social services and psychiatric care.

When exploring ethical approaches and respect for rights, the recognition of social inequalities in access to care and the importance of protecting the rights of vulnerable individuals were recurring themes in the discussions. The need for a respectful and non-stigmatizing approach, considering the various obstacles individuals face, is a fundamental principle already addressed in the literature. Respecting human rights in psychiatric care is a central aspect of the work of Kelly (2015), which emphasizes the importance of offering non-coercive, dignity-based care. The focus on individual rights, including their right to equitable access to psychiatric care, should also be a key issue in public health policies, as outlined by the World Health Organization (2021).

Concerning specific populations and associated challenges, the results primarily highlight the vulnerabilities related to homelessness. While the literature identifies various at-risk groups, such as LGBTQ+ youth or women in precarious situations (Côté et al., 2023; Bretherton, 2020), the discussions in this study were largely centered on homelessness and related phenomena. Experts emphasized the prevalence of hidden homelessness among women, a phenomenon well-documented in the literature (Andermann et al., 2021). Although homelessness, migration, ethnic minority status, LGBTQ+ identity, and mental health disorders all contribute to social disadvantages, the nature and causes of these vulnerabilities vary and should be acknowledged. However, the presented data do not allow us to draw strong conclusions about the necessary adaptations for LGBTQ-friendly or culturally appropriate care. That being said, the study provided valuable insights into the trajectories and causes of homelessness among both adults and youth. In particular, it emphasized key contributing factors such as stigma, poverty, and family breakdown, findings that are also well-documented in the literature by Mejia-Lancheros et al. (2020).

When considering intervention and support models, proximity intervention models, like the ÉQIIP SOL for youth and PRISM, have been identified throughout the discussions as very effective in supporting individuals in their stabilization process, facilitating access and engagement. Literature on proximity interventions and post-hospitalization follow-up supports these practices, highlighting their effectiveness in maintaining individuals’ stability after hospitalization and supporting their reintegration (Tsemberis et al., 2004). The Housing First model is also mentioned as a successful example of integrating psychiatric care and housing for homeless populations (Aubry et al., 2016).

When exploring innovation and evolution of practices, this study revealed a willingness to adopt new practices, integrate research into interventions and integrate community center internships for residents so that they can develop their structural competences. Participants believe that those changes would help meet the changing needs of vulnerable populations. This aligns with observations in the literature, where innovation and service adaptation are seen as essential to meet contemporary challenges in the mental health sector (Piat & Sabetti, 2012). Adapting services to the realities of vulnerable populations allows better responses to the complex and evolving needs of individuals in precarious situations. 

Key recommendations emerged from this study to improve care for vulnerable populations. First, strengthening intersectoral partnerships is essential; promoting collaboration between health and social service institutions—such as hospitals and community organizations—ensures comprehensive and consistent care. Participants highlighted the critical role of these partnerships in supporting those populations and emphasized the need for trust and effective communication between teams and organizations. Second, developing personalized approaches tailored to specific needs is crucial. This can be carried out by strengthening proximity care models. Mental health workers in this study recognize that programs like home hospitalization and PRISM are humane and effective tools for supporting individuals during stabilization processes and ensure better access and sustained engagement in care. Third, we believe it is essential for the government to invest in the construction of affordable housing so that the Housing First model can be realistically implemented. Finally, implementing awareness and training programs for mental health professionals is necessary to combat discrimination and stigma. The recognition of social inequalities in access to care and the importance of protecting the rights of vulnerable individuals were themes addressed by the participants. This could start with integrating mandatory community center internships for psychiatry residents.

Confirmation bias may have led researchers to emphasize certain themes that aligned with their initial hypotheses. Similarly, perception bias could have influenced participants, as their professional and personal experiences might shape a partial view of the reality faced by vulnerable populations. Furthermore, the complexity of data representation presents a challenge, because themes simplify the data but can overlook nuanced or contradictory participant experiences. Information can be lost when aggregating data into themes. Finally, while the study aimed to address a plurality of vulnerabilities, the discussions predominantly focused on homelessness.

## 5. Conclusions

This qualitative study highlights major challenges related to access to care, stigma, and the need for personalized and intersectoral approaches for specific populations. The findings emphasize the importance of housing stability, service integration, and respect of individuals’ rights in the care trajectory of patients. Based on these results, we recommend improving intersectoral collaboration, extending flexible care models, and integrating more clinical exposition to marginalized populations in the community sector. The next steps include validating these recommendations in practical settings and evaluating the impact of the proposed interventions on targeted populations. The results did not extensively address changes in the teaching of psychiatry residents that would better prepare them to work with specific populations and clienteles, but the necessary changes in pedagogy could be explored in a subsequent study.

## Figures and Tables

**Table 1:** Course schedule and topics covered in each section.

Time	Activities	Topics Covered
First half of the morning	Class on Youth homelessness by Psychiatrist C	Class addressing causes of youth homelessness, intervention, and support models for youth experiencing homelessness and the importance of collaboration with community organizations
Second half of the morning	Class on homelessness, home-based hospitalization, and the importance of proximity-based intervention by Psychiatrist B	Class addressing homelessness, crisis intervention methods, home-based hospitalization, proximity-based intervention, housing accesss, institutional changes, and service adaptation needs, as well as respect and dignity for homeless individuals
Afternoon (Group 1)	Visit of Old Brewery Mission—Webster Pavilion (shelter for men) with discussions with mental health workers from the Intensive Support for Homelessness Program (SII) and the Project for Reaffiliation in Homelessness and Mental Health (PRISM). Half of the psychiatry residents visited with Psychiatrist B.	Discussions focusing on flexible approaches, therapeutic opportunity windows, harm reduction strategies, collaboration with community organizations, rights of vulnerable individuals in psychiatric care, and the importance of dignity for homeless individuals.
Afternoon (Group 2)	Visit of Old Brewery Mission—Patricia Mackenzie Pavilion (shelter for women) with discussions with mental health workers from the Project for Reaffiliation in Homelessness and Mental Health (PRISM). The other half of the psychiatry residents visited with Psychiatrist A.	Discussions focusing on women in precarious situations flexible approaches, therapeutic opportunity window, rights of vulnerable individuals in psychiatric care, collaboration with community organizations, housing access, and the importance of housing and the importance of dignity for homeless individuals.
End of the afternoon	Group debrief on the day’s learning	Discussions focusing on innovation and evolution of practices, the importance of collaboration with community organizations as well as institutional changes and service adaptation needs.

**Table 2:** Overview of themes and sub-themes related to adaptations in psychiatric approaches for vulnerable populations.

Themes	Sub-Themes	Examples
**Barriers and Needs of Vulnerable Populations**	Specific psychosocial needs of vulnerable populations	“Therefore, we (*the PRISM team*) want to remove obstacles to life planning: health problems, mental illness, and symptoms are obstacles”.
	Challenges in housing access and the importance of housing	“If they have housing instability … we can “offer them medication”, as they say, but health is not their immediate concern”.
	Challenges in accessing healthcare and services	“But often, they (*young people in situations of homelessness or precariousness*) don’t want to know anything (*about healthcare*) for three months, and then they realize the consequences, and then they would like to get help, but the way the system is set up, when your file is closed, it means you find services, and then, well, you know the rest, they don’t have a health insurance card, they are unreachable, they don’t know where to knock, etc”.
	Impact of poverty and stigma	“Be careful with your biases or predictions; we have unfavorable biases in our prognosis because of these factors (*stigma*)”.
	Experiences of violence and abuse	“Then there are the users who build up debts with people who use violent methods to collect what they’re owed”.
**Psychiatric Interventions and Flexible Approaches**	Approaches tailored to homeless youth	“We (*the ÉQIIP SOL team*) provide care—care that is specifically oriented toward helping individuals exit homelessness”.
	Approaches tailored to homeless adults	“We (*the PRISM team*) issue identification if they don’t have any, then we arrange income if they don’t have any, medical evaluations, treatment, fill out paperwork, and we make housing arrangements, then make the referral”.
	Crisis intervention methods and home-based hospitalization	“We (*the medical team*) put ourselves in our patients’ shoes: What would we want, how would we want to experience our acute psychiatric episode? … behind a magnetic door, with an isolation room, restraints, hospital food… or at home, in our slippers, with our loved ones around?”
	Substance harm reduction strategies	“They continue their life seeking substances and so on… but *because of the medication they recieve* they’re not arrested by the police three times a week with charges because they became aggressive”.
	Therapeutic opportunity windows	“Sometimes, it’s 15 min between the time they (*patients*) arrive, we don’t know them, we talk to them, and they agree to an injection; we administer it”.
**Collaboration with Community Organizations**	Role of organizations in homelessness support	“Diogène is an excellent partner, and the reason for this is that they truly embody what Housing First is”.
	Community partnerships for psychiatric care	“Once they (*psychiatry residents*) understood that all the responsibility for deciding on the patient’s treatment didn’t fall solely on them, and that it’s completely… when I talk about everyone lifting the car with two fingers by each applying equal strength, that’s the strength of this type of team—it becomes an addictive feeling”.
	Challenges and successes in intersectoral collaboration	“There are cases of discharging homeless people with significant mental health issues back to emergency shelters, in conditions that are impossible to manage in these environments, without any communication or liaison, which is senseless”.
	Importance of trust between teams and organizations	“When they (*community outreach workers*) talk about sending a patient to the hospital and we (*the medical team*) quickly send them back… it’s just a reminder that it’s essential to work as a team”.
**Ethical Approach and Respect for Rights**	Respect and dignity for homeless individuals	“It’s not ideal for them to camp in public spaces, but at the same time, they form a kind of community, providing forms of protection, like overdose prevention”.
	Awareness of social inequalities	“So, a homeless person doesn’t have a RAMQ card (*help assurance card*), doesn’t have any identification papers—you can prescribe the best medicine in the world, but at the pharmacy, they won’t be able to get the medication”.
	Rights of vulnerable individuals in psychiatric care	“And when everyone is on an equal footing (*like in the community setting*), the patient is also on an equal footing with us”.
**Specific Populations and Associated Challenges**	LGBTQ+ youth in family conflict	“This (*discrimination within the family*) doesn’t mean that we shouldn’t work with families—we do work closely with them—but we need to be sensitive to this”.
	Individuals without family networks	“There’s no one there to notice subtle signs and become concerned about the onset of illness”.
	Youth homelessness: causes and trajectories	“More typically, what we used to see, and we still see sometimes, is that, you know, they’d finish with youth protection services, and then they’d arrive with a garbage bag, with their stuff inside, at the door of the Refuge des Jeunes”.
	Adult homelessness: causes and trajectories	“There are people who have had breaks in their lives; they had a normal, good life, and then suddenly, financial disaster, a breakup, an illness episode—something happens, and everything collapses”.
	Women in precarious situations	“It’s always been about two-thirds men (*in the homeless population*), one-third women, but hidden homelessness is much more common among women—women will stay with men who may exploit them sexually or in other ways”.
	**Migrant populations’ struggles**	“Before even supporting them in stabilization efforts, their immigration status takes precedence—it’s even more crucial for stabilizing their mental state than interventions like medication”.
**Intervention and Support Models**	ÉQIIP SOL model for youth	“So, the support workers have half the caseloads of others, and they also naturally engage with community organizations—since they have smaller caseloads, they’re much more able to do close-range interventions”.
	Importance of proximity-based intervention	“When you’re in the community, you’re in constant contact with people, so you can suggest, be told no, and say, “At least you know we exist, you know what we do”. … and occasionally, if we see you, we’ll check in to see how things are going so that, on the day, the moment when they say, “Hey, my life’s a mess, I’d maybe like to change something,” we’re there”.
	Intensive post-hospitalization follow-up	“In fact, she just reintegrates into PRISM (*PRISM user in post-hospitalization*)”.
	Training and integration of diverse services (e.g., housing, mental health, social services)	“But for social workers and other support workers (*at PRISM*), the scope of practice requires quick responses, flexibility, roles in assessment, program integration, referrals, and liaising”.
**Innovation and Evolution of Practices**	Development of new, adapted resources and practices	“And we give ourselves 6, 8, 10, 12 weeks (*at PRISM*)—a set number of weeks to handle identification papers, income, medical and psychiatric needs assessment, introduce treatment, work on stable, permanent housing, and connect the person to the services they need”.
	Influence of research and documentation on intervention impact	“And we can see, actually, in the second study I didn’t show you, that housing improves slightly before symptoms do”.
	Institutional changes and service adaptation needs	“Because they (*the government*) wanted to develop PRISMs, but I said there’s no point in developing PRISMs and SIIs if there’s nothing (*no housing option*) afterward… It’s like an assembly line—if the assembly line is blocked, we won’t be able to refer patients anywhere”.
	Evaluation and feedback on clinical and community approaches	“It reminds us of the importance of understanding the trajectory of what we want—not just understanding it but also questioning if our current structure has inconsistencies and highlighting them, so we can keep working for a smooth process where everyone can transition from one stage to the next in a meaningful way”.

## Data Availability

The data presented in this study are available on request from the corresponding author. The data are not publicly available due to participants’ privacy.

## References

[B1-ejihpe-15-00030] Andermann A., Mott S., Mathew C. M., Kendall C., Mendonca O., Harriott D., McLellan A., Riddle A., Saad A., Iqbal W., Magwood O., Pottie K. (2021). Evidence-informed interventions and best practices for supporting women experiencing or at risk of homelessness: A scoping review with gender and equity analysis. Health Promotion and Chronic Disease Prevention in Canada: Research, Policy and Practice.

[B2-ejihpe-15-00030] Aubry T., Goering P., Veldhuizen S., Adair C. E., Bourque J., Distasio J., Latimer E., Stergiopoulos V., Somers J., Streiner D. L., Tsemberis S. (2016). A multiple-city RCT of Housing first with assertive community treatment for homeless Canadians with serious mental illness. Psychiatric Services.

[B3-ejihpe-15-00030] Barker L. C., Lee-Evoy J., Butt A., Wijayasinghe S., Nakouz D., Hutcheson T., McCarney K., Kaloty R., Vigod S. N. (2022). Delivering collaborative mental health care within supportive housing: Implementation evaluation of a community-hospital partnership. BMC Psychiatry.

[B4-ejihpe-15-00030] Baxter A. J., Tweed E. J., Katikireddi S. V., Thomson H. (2019). Effects of Housing First approaches on health and well-being of adults who are homeless or at risk of homelessness: Systematic review and meta-analysis of randomised controlled trials. Journal of Epidemiology and Community Health.

[B5-ejihpe-15-00030] Bhui K., Morgan N. (2007). Effective psychotherapy in a racially and culturally diverse society. Advances in Psychiatric Treatment.

[B6-ejihpe-15-00030] Bretherton J. (2020). Women’s experiences of homelessness: A longitudinal study. Social Policy and Society.

[B7-ejihpe-15-00030] Castillo E. G., Ijadi-Maghsoodi R., Shadravan S., Moore E., Mensah M. O., Docherty M., Aguilera Nunez M. G., Barcelo N., Goodsmith N., Halpin L. E., Morton I., Mango J., Montero A. E., Rahmanian Koushkaki S., Bromley E., Chung B., Jones F., Gabrielian S., Gelberg L., Greenberg J. M., Wells K. B. (2019). Community interventions to promote mental health and social equity. Current Psychiatry Reports.

[B8-ejihpe-15-00030] Castle D. J., Lusicic A., Petrakis M., Bhugra D., Bhui K., Wong S. Y. S., Gilman S. E. (2018). Early intervention in psychiatry. Oxford textbook of public mental health.

[B9-ejihpe-15-00030] Chomczynski P. (2008). QDA MINER—The mixed method solution for qualitative analysis by provalis research. Qualitative Sociology Review.

[B10-ejihpe-15-00030] Chun Tie Y., Birks M., Francis K. (2019). Grounded theory research: A design framework for novice researchers. SAGE Open Medicine.

[B11-ejihpe-15-00030] Cooke B. K., Garvan C., Hobbs J. A. (2013). Trends in performance on the psychiatry resident-in-training examination (PRITE^®^): 10 years of data from a single institution. Academic Psychiatry: The Journal of the American Association of Directors of Psychiatric Residency Training and the Association for Academic Psychiatry.

[B12-ejihpe-15-00030] Corrigan P. W., Al-Khouja M. A. (2018). Three agendas for changing the public stigma of mental illness. Psychiatric Rehabilitation Journal.

[B13-ejihpe-15-00030] Côté P.-B., Frésard L., Blais M. (2023). ‘I didn’t want to be noticed’: Discrimination and violence among LGBTQ+ youth experiencing homelessness. Journal of LGBT Youth.

[B14-ejihpe-15-00030] Daniels S. I., Cheng H., Gray C., Kim B., Stave C. D., Midboe A. M. (2022). A scoping review of implementation of health-focused interventions in vulnerable populations. Translational Behavioral Medicine.

[B15-ejihpe-15-00030] Farkas M., Anthony W. A., Montenegro R., Gayvoronskaya E., Messich J., Botbol M., Christodolou G., Cloninger R., Salloum I. (2016). Person-centered psychiatric rehabilitation. Person-centered psychiatry.

[B16-ejihpe-15-00030] Fennig M. (2021). Cultural adaptations of evidence-based mental health interventions for refugees: Implications for clinical social work. The British Journal of Social Work.

[B17-ejihpe-15-00030] Galea S., Tracy M., Hoggatt K. J., Dimaggio C., Karpati A. (2011). Estimated deaths attributable to social factors in the United States. American Journal of Public Health.

[B18-ejihpe-15-00030] Hatzenbuehler M. L., Bellatorre A., Lee Y., Finch B. K., Muennig P., Fiscella K. (2018). Corrigendum to “Structural stigma and all-cause mortality in sexual minority populations” [Soc. Sci. Med. 103 (2014) 33–41]. Social Science & Medicine.

[B19-ejihpe-15-00030] Hwang S. W., Aubry T., Palepu A., Farrell S., Nisenbaum R., Hubley A. M., Klodawsky F., Gogosis E., Hay E., Pidlubny S., Dowbor T., Chambers C. (2011). The health and housing in transition study: A longitudinal study of the health of homeless and vulnerably housed adults in three Canadian cities. International Journal of Public Health.

[B20-ejihpe-15-00030] Kelly B. D. (2015). Human rights in psychiatric practice: An overview for clinicians. BJPsych Advances.

[B21-ejihpe-15-00030] Kirmayer L. J., Pedersen D. (2014). Toward a new architecture for global mental health. Transcultural Psychiatry.

[B22-ejihpe-15-00030] Kloos B., Hill J., Thomas E., Case A., Scott V., Wandersman A. (2021). Community psychology: Linking individuals and communities.

[B23-ejihpe-15-00030] Lincoln Y. S., Guba E. G. (1985). Naturalistic inquiry.

[B24-ejihpe-15-00030] Magwood O., Salvalaggio G., Beder M., Kendall C., Kpade V., Daghmach W., Habonimana G., Marshall Z., Snyder E., O’Shea T., Lennox R., Hsu H., Tugwell P., Pottie K. (2020). The effectiveness of substance use interventions for homeless and vulnerably housed persons: A systematic review of systematic reviews on supervised consumption facilities, managed alcohol programs, and pharmacological agents for opioid use disorder. PLoS ONE.

[B25-ejihpe-15-00030] Marsh J. L., O’Mallon M., Stockdale S., Potter D. R. (2020). Caring for vulnerable populations during a pandemic: Literature review. International Journal of Caring Sciences.

[B26-ejihpe-15-00030] McGorry P. D., Mei C., Dalal N., Alvarez-Jimenez M., Blakemore S. J., Browne V., Dooley B., Hickie I. B., Jones P. B., McDaid D., Mihalopoulos C., Wood S. J., El Azzouzi F. A., Fazio J., Gow E., Hanjabam S., Hayes A., Morris A., Pang E., Killackey E. (2024). The lancet psychiatry commission on youth mental health. Lancet Psychiatry.

[B27-ejihpe-15-00030] Mejia-Lancheros C., Lachaud J., O’Campo P., Wiens K., Nisenbaum R., Wang R., Hwang S. W., Stergiopoulos V. (2020). Trajectories and mental health-related predictors of perceived discrimination and stigma among homeless adults with mental illness. PLoS ONE.

[B28-ejihpe-15-00030] Metzl J. M., Hansen H. (2014). Structural competency: Theorizing a new medical engagement with stigma and inequality. Social Science & Medicine.

[B29-ejihpe-15-00030] Olfson M., Stroup T. S., Huang C., Wall M. M., Crystal S., Gerhard T. (2021). Suicide risk in medicare patients with schizophrenia across the life span. JAMA Psychiatry.

[B30-ejihpe-15-00030] Padgett D. K. (2020). Homelessness, housing instability and mental health: Making the connections. BJPsych Bulletin.

[B31-ejihpe-15-00030] Piat M., Sabetti J. (2012). Recovery in Canada: Toward social equality. International Review of Psychiatry.

[B32-ejihpe-15-00030] Pinkhover A., Celata K., Baker T., Chatterjee A., Lunze K. (2024). Mobile addiction treatment and harm reduction services as tools to address health inequities: A community case study of the Brockton neighborhood health center mobile unit. Frontiers in Public Health.

[B33-ejihpe-15-00030] Russolillo A., Moniruzzaman A., Carter M., Raudzus J., Somers J. M. (2023). Association of homelessness and psychiatric hospital readmission-a retrospective cohort study 2016–2020. BMC Psychiatry.

[B34-ejihpe-15-00030] Scott P. (2017). The SAGE encyclopedia of communication research methods.

[B35-ejihpe-15-00030] Stergiopoulos V., Gozdzik A., O’Campo P., Holtby A. R., Jeyaratnam J., Tsemberis S. (2014). Housing first: Exploring participants’ early support needs. BMC Health Services Research.

[B36-ejihpe-15-00030] Sudak D. M., DeJong S. M., Bailey B., Rohrbaugh R. M. (2020). Training psychiatrists to achieve mental health equity. The Psychiatric Clinics of North America.

[B37-ejihpe-15-00030] Tsemberis S., Ellen I. G., O’Flaherty B. (2010). Housing First: Ending homelessness, promoting recovery, and reducing costs. How to house the homeless.

[B38-ejihpe-15-00030] Tsemberis S., Gulcur L., Nakae M. (2004). Housing first, consumer choice, and harm reduction for homeless individuals with a dual diagnosis. American Journal of Public Health.

[B39-ejihpe-15-00030] Uhlhaas P. J., Davey C. G., Mehta U. M., Shah J., Torous J., Allen N. B., Avenevoli S., Bella-Awusah T., Chanen A., Chen E. Y. H., Correll C. U., Do K. Q., Fisher H. L., Frangou S., Hickie I. B., Keshavan M. S., Konrad K., Lee F. S., Liu C. H., Wood S. J. (2023). Towards a youth mental health paradigm: A perspective and roadmap. Molecular Psychiatry.

[B40-ejihpe-15-00030] Wang J. Z., Mott S., Magwood O., Mathew C., Mclellan A., Kpade V., Gaba P., Kozloff N., Pottie K., Andermann A. (2019). The impact of interventions for youth experiencing homelessness on housing, mental health, substance use, and family cohesion: A systematic review. BMC Public Health.

[B41-ejihpe-15-00030] Williams R., Jenkins D. A., Ashcroft D. M., Brown B., Campbell S., Carr M. J., Cheraghi-Sohi S., Kapur N., Thomas O., Webb R. T., Peek N. (2020). Diagnosis of physical and mental health conditions in primary care during the COVID-19 pandemic: A retrospective cohort study. The Lancet Public Health.

[B42-ejihpe-15-00030] World Health Organization (2021). Guidance on community mental health services: Promoting person-centred and rights-based approaches.

